# Quasi‐Homoepitaxial Growth of Highly Strained Alkali‐Metal Ultrathin Films on Kagome Superconductors

**DOI:** 10.1002/advs.202309003

**Published:** 2024-06-03

**Authors:** Takemi Kato, Kosuke Nakayama, Yongkai Li, Zhiwei Wang, Katsuaki Sugawara, Kiyohisa Tanaka, Takashi Takahashi, Yugui Yao, Takafumi Sato

**Affiliations:** ^1^ Department of Physics Graduate School of Science Tohoku University Sendai 980–8578 Japan; ^2^ Centre for Quantum Physics Key Laboratory of Advanced Optoelectronic Quantum Architecture and Measurement (MOE) School of Physics Beijing Institute of Technology Beijing 100081 P. R. China; ^3^ Beijing Key Lab of Nanophotonics and Ultrafine Optoelectronic Systems Beijing Institute of Technology Beijing 100081 P. R. China; ^4^ Material Science Center Yangtze Delta Region Academy of Beijing Institute of Technology Jiaxing 314011 P. R. China; ^5^ Precursory Research for Embryonic Science and Technology (PRESTO) Japan Science and Technology Agency (JST) Tokyo 102‐0076 Japan; ^6^ Advanced Institute for Materials Research (WPI‐AIMR) Tohoku University Sendai 980–8577 Japan; ^7^ UVSOR Synchrotron Facility Institute for Molecular Science Okazaki 444–8585 Japan; ^8^ School of Physical Sciences The Graduate University for Advanced Studies (SOKENDAI) Okazaki 444–8585 Japan; ^9^ Center for Science and Innovation in Spintronics (CSIS) Tohoku University Sendai 980–8577 Japan; ^10^ International Center for Synchrotron Radiation Innovation Smart (SRIS) Tohoku University Sendai 980–8577 Japan; ^11^ Mathematical Science Center for Co‐creative Society (MathCCS) Tohoku University Sendai 980–8578 Japan

**Keywords:** alkali metals, angle‐resolved photoemission spectroscopy, homoepitaxy, kagome superconductors, quantum‐well states, thin films

## Abstract

Applying lattice strain to thin films, a critical factor to tailor their properties such as stabilizing a structural phase unstable at ambient pressure, generally necessitates heteroepitaxial growth to control the lattice mismatch with substrate. Therefore, while homoepitaxy, the growth of thin film on a substrate made of the same material, is a useful method to fabricate high‐quality thin films, its application to studying strain‐induced structural phases is limited. Contrary to this general belief, here the quasi‐homoepitaxial growth of Cs and Rb thin films is reported with substantial in‐plane compressive strain. This is achieved by utilizing the alkali‐metal layer existing in bulk crystal of kagome metals *A*V_3_Sb_5_ (*A* = Cs and Rb) as a structural template. The angle‐resolved photoemission spectroscopy measurements reveal the formation of metallic quantum well states and notable thickness‐dependent quasiparticle lifetime. Comparison with density functional theory calculations suggests that the obtained thin films crystalize in the face‐centered cubic structure, which is typically stable only under high pressure in bulk crystals. These findings provide a useful approach for synthesizing highly strained thin films by quasi‐homoepitaxy, and pave the way for investigating many‐body interactions in Fermi liquids with tunable dimensionality.

## Graphical abstract

A compressed Cs layer embedded in bulk crystal of kagome superconductor, CsV_3_Sb_5_, is demonstrated to be an appropriate substrate to fabricate Cs thin films with the face‐centered cubic structure, which is typically realized only under high pressure in bulk Cs. The present results provide a useful guiding principle for introducing significant lattice strain in the homoepitaxial technique.

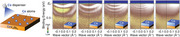

## 1 Introduction

The epitaxial growth of thin films on substrate is a fundamental technique crucial for advancing both basic science and industrial applications. One of its great advantages is the capability to apply lattice strain to the film, which can stabilize structural phases that are unstable under ambient conditions and/or drastically alter the physical properties such as optical absorption, superconductivity, magnetism, and topological phase.^[^ ^1^, ^2^, ^3^, ^4^, ^5^, ^6^ ^]^ Lattice strain is typically introduced by the lattice mismatch between the film and the substrate, necessitating heteroepitaxy. However, heteroepitaxy sometimes faces challenges, such as difficulties in producing high‐quality films due to the intermixing between the film and substrate materials and the defect formation originating from the significant lattice mismatch. To achieve high purity in the film, homoepitaxy is a promising approach, as demonstrated in the production of epitaxial silicon wafer. Nevertheless, homoepitaxy is generally unable to introduce lattice strain due to the perfect lattice match. Here, we report an example of overcoming such an essential challenge to obtain highly strained thin films by quasi‐homoepitaxy.

**Figure 1:**
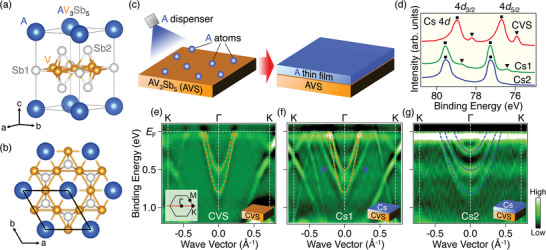
a,b) Crystal structure of *A*V_3_Sb_5_ (AVS) and its top view, respectively. The graphics were created by VESTA.^[^ ^49^ ^]^ Black lines represent the unit cell of AVS. c) Schematics of alkali‐metal deposition and growth of an alkali‐metal thin film on AVS substrate. d) ARPES spectra in the Cs‐4*d* core‐level region for pristine CsV_3_Sb_5_ (CVS; red) and Cs‐dosed CVS (Cs1; green, Cs2; blue). Triangles and squares indicate the peak positions of bulk‐ and surface‐derived Cs‐4*d* core levels, respectively. e–g) Second‐derivative ARPES intensity plots for CVS, Cs1, and Cs2 samples, respectively, measured along the ΓK cut in the first Brillouin zone shown by red line in the inset of (e) at *hν* = 56 eV. Orange and blue dashed curves are a guide to trace the electron‐band dispersions of CVS and the quantum‐well states, respectively.

We chose *A*V_3_Sb_5_ (AVS; *A* = K, Rb, Cs) as a substrate, which is a recently discovered quantum material family exhibiting superconductivity (*T*_c_ = 0.9–2.5 K) and the 2 × 2 × 2 or 2 × 2 × 4 charge‐density wave (CDW) (*T*_CDW_ = 78–103 K).^[^ ^7^, ^8^, ^9^, ^10^, ^11^, ^12^, ^13^, ^14^, ^15^, ^16^, ^17^, ^18^, ^19^, ^20^, ^21^ ^]^ As shown in **Figure** **1**a,b, AVS crystallizes in a layered structure consisting of V‐based kagome‐lattice layer with hexagonally arranged Sb atoms (Sb1), graphene‐like honeycomb Sb layer (Sb2), and hexagonal alkali‐metal layer (A). AVS crystal cleaves between the alkali metal and Sb2 layers due to their weak bonding. Consequently, the cleaved surface is terminated either by alkali‐metal or Sb2 atoms, as observed by scanning tunneling microscopy.^[^ ^14^, ^15^, ^16^, ^17^, ^18^, ^19^, ^20^, ^21^ ^]^ Intriguingly, there is growing experimental evidence that the AVS surface hosts peculiar properties, such as the formation of 4 × 1 CDW, pair density wave, unusual vortex core states, and polar charge accumulation.^[^ ^15^, ^16^, ^17^, ^18^, ^19^, ^20^, ^21^, ^22^, ^23^ ^]^ Some of these properties exhibit a strong surface‐termination dependence.^[^ ^15^, ^16^, ^17^, ^18^, ^19^, ^20^, ^21^, ^22^, ^23^ ^]^ Moreover, the deposition of Cs atoms on the CsV_3_Sb_5_ (CVS) surface results in the tuning of surface charge accumulation and the suppression of the CDW order.^[^ ^24^ ^]^ These results demonstrate that the tuning of alkali‐metal coverage at the AVS surface is a promising approach to control and explore the novel physical properties.

In this study, we attempted to realize and investigate the novel phase with a high alkali‐metal coverage well beyond the endpoint of the CDW phase. To achieve this, we enhanced the adsorption rate of alkali‐metal atoms by depositing them onto the AVS surface at low temperatures in contrast to the previous work where the alkali‐metal deposition was carried out at room temperature.^[^ ^24^ ^]^ However, as a result, we discovered the unexpected formation of single‐crystalline Cs (and Rb) thin films, as schematically illustrated in Figure 1c. This thin film formation is regarded as quasi‐homoepitaxial growth on the Cs‐terminated surface of CVS. Furthermore, due to the shorter atomic distance between adjacent Cs atoms in CVS compared to that in bulk Cs, the obtained thin films appear to be substantially strained, forming the face‐centered cubic (fcc) structure unstable in bulk Cs at ambient pressure. This result contrasts with the conventional knowledge that large strain is hardly introduced by homoepitaxy. By using angle‐resolved photoemission spectroscopy (ARPES), we have investigated the evolution of electronic states as a function of film thickness. This study is consequently valuable for the investigation of quantum‐well states (QWSs), which have been predicted and observed to exhibit various physical properties as tailor‐made 2D electron systems.^[^ ^25^, ^26^, ^27^, ^28^, ^29^, ^30^ ^]^ Thus far, most QWS studies have concentrated on complex systems such as strongly correlated electron systems and topological materials, with little attention to the fundamental behavior of QWSs in very simple systems like alkali metals. The present study establishes one of the simplest prototype QWS systems and opens the way for comparative studies with complex systems.

## 2 Results and Discussion

### 2.1 Fabrication of Cs Thin Films and Characterization of Band Structure

First, we present the evolution of the Cs‐4*d* core level upon Cs deposition onto CVS (Figure 1d). In pristine CVS before Cs deposition (red curve), each spin‐orbit satellite (*d*_5/2_ and *d*_3/2_) consists of two peaks. The peaks with stronger intensity at higher binding energies (*E*_B_’s), indicated by square, originate from Cs atoms at the topmost surface, whereas the peaks with weaker intensity at lower *E*_B_’s (triangle) are associated with bulk Cs atoms beneath the V‐Sb layer.^[^ ^22^, ^23^ ^]^ The stronger intensity of the surface component suggests that the cleaved surface probed here is predominantly Cs‐terminated. In the sample after Cs deposition at *T* = 20 K (Cs1 sample; green), all the peaks shift to higher *E*_B_’s. In addition, the bulk components diminish, indicating the formation of a Cs overlayer that hinders photoemission from the bulk crystal portion deep from the surface. In the sample after additional Cs deposition (Cs2 sample; blue), only the peaks from surface Cs are observed, with no detectable bulk Cs components.

To elucidate the evolution of the band structure near the Fermi level (*E*_F_) in response to the change in the core level spectrum, we plot in Figure 1e–g the second‐derivative ARPES intensity measured along the ΓK cut of the hexagonal Brillouin zone for the pristine, Cs1, and Cs2 samples. In the pristine sample (Figure 1e), as reported in previous ARPES studies,^[^ ^31^, ^32^, ^33^, ^34^, ^35^, ^36^, ^37^, ^38^, ^39^ ^]^ there are two electron bands with band bottom at *E*_B_ ≈ 0.5 and 0.8 eV, respectively, at the Γ point (indicated by orange dashed curves). In addition, there are Dirac‐cone‐like bands near the K point and linearly dispersive bands between the Γ and K points. In the Cs1 sample (Figure 1f), apart from these CVS‐derived bands, another electron band bottomed at ≈0.6 eV is identified around the Γ point (highlighted by blue arrows). In the Cs2 sample (Figure 1g), the intensity of the CVS‐derived bands is markedly suppressed, and three electron bands bottomed at *E*_B_ ≈0.3, 0.5, and 0.8 eV, respectively, (blue dashed curves) are clearly observed. These electron bands exhibit a similar velocity to that of the electron band emerging in the Cs1 sample, suggesting a common origin; most likely, these electron bands are metallic QWSs originating from the single‐crystalline Cs thin film.

### 2.2 Thickness‐Dependent Quantum Confinement

**Figure 2:**
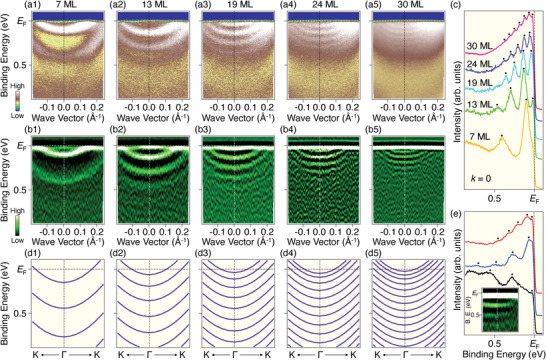
a1–a5) ARPES intensity at *T* = 20 K for Cs‐deposited CVS measured along the ΓK cut. The thickness is estimated to be 7, 13, 19, 24, and 30 monolayers (MLs), respectively. b1–b5) Second derivative intensity of (a1)–(a5). c) ARPES spectra measured at the Γ point for (a1)–(a5). d1–d5) Calculated band structure along the ΓK cut for 7‐, 13‐, 19‐, 24‐, and 30‐ML fcc Cs(111), respectively. e) ARPES spectra measured at the Γ point for Rb‐deposited RbV_3_Sb_5_. Deposition time increases in the order of black, blue, and red. Inset shows the second‐derivative ARPES intensity measured at *T* = 20 K along the ΓK cut. Black dots in (c) and (e) indicate the peak positions in ARPES spectra.

To confirm the QWS nature of the newly emerging electron bands, we performed a systematic study on the evolution of the band structure as a function of film thickness. For this sake, we conducted multiple cycles of Cs deposition and ARPES measurements while keeping the deposition rate and time constant at each deposition, ensuring the consistent increase in the overlayer thickness per cycle. **Figure** **2**a1–a5 displays plots of ARPES intensity around the Γ point measured after 1–5 cycles of Cs deposition. Figure 2b1–b5 displays the corresponding second‐derivative intensity. The number of the electron bands is at least two after the first deposition (Figure 2a1,b1) and gradually increases up to more than five after the fifth deposition (Figure 2a5,b5). In addition, the energy separation between the electron bands systematically narrows from ≈300 meV (Figure 2a1,b1) to ≈50 meV (Figure 2a5,b5), as also evident from a comparison of the raw ARPES spectra at the Γ point in Figure 2c (the number of Cs‐deposition cycle increases from bottom to top). These changes are consistent with the behavior typically anticipated for QWSs when increasing the width of a quantum well.^[^ ^25^, ^26^, ^27^, ^28^, ^29^, ^30^ ^]^ Since the energy separation between QWSs is a measure of the film thickness, we performed band structure calculations for free‐standing Cs slabs with varying the thickness, and compared with the experimental band structures to estimate the film thickness, as described in detail in Supporting Information. Figure 2d1–d5 shows the calculated results that exhibit the best match of the energy separation with the experimental data. In these calculations, we assumed the fcc structure (the reason for this choice will be discussed later). Notably, the calculated band structure well reproduces not only the energy separation but also the velocity of the experimental band dispersion, supporting the QWS nature of the observed electron bands. Based on this good agreement, the film thickness in Figure 2a1–a5 is determined to be 7, 13, 19, 24, and 30 monolayers (MLs; equal to atomic layers), respectively. This suggests that the film thickness was proportional to the number of deposition cycle (*n*), as ≈6*n*+1. It is noted that the thinnest film obtained so far is 7 ML, which corresponds to two unit‐cell thick. Although we tried to obtain thinner films by reducing the deposition time and rate, in this case, clear QWSs were not observed by ARPES. This result implies that achieving uniformity over a large lateral size comparable to the beam spot of the ARPES measurement might be difficult in extremely thin films.

Besides Cs thin films on CVS, we fabricated Rb thin films on RbV_3_Sb_5_ (RVS). A representative second‐derivative ARPES intensity plot in the inset of Figure 2e shows the appearance of several electron bands centered at the Γ point, suggesting the formation of QWSs. In Figure 2e, ARPES spectra taken at the Γ point for samples with increasing deposition cycles (in the order of black, blue, and red) reveal an increase in the number of QWSs and a narrowing of their energy separation, as observed in Cs QWSs. We also tried low‐temperature K deposition on KV_3_Sb_5_ (KVS), but were unable to obtain single‐crystalline films in contrast to the Cs and Rb cases. We will revisit this issue later.

### 2.3 Analysis of Structural Stability by Density Functional Theory

**Figure 3:**
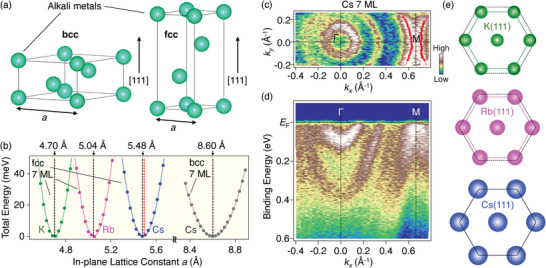
a) Crystal structure of alkali metals with body‐centered cubic (bcc; left) and fcc (right) structure. One unit cell is shown for each. b) Calculated total energy as a function of in‐plane lattice constant *a* [defined in (a)] for 7‐ML fcc K (green), Rb (magenta), Cs (blue), and bcc Cs (gray). Solid lines are the results of numerical fittings by quadratic functions. The lowest energy is achieved at *a* = 4.70 Å for fcc K, 5.04 Å for fcc Rb, 5.48 Å for fcc Cs, and 8.60 Å for bcc Cs (indicated by black dashed lines). Red dashed line indicates the lattice constant of bulk CVS (*a* = 5.49 Å). c) ARPES intensity at *E*_F_ plotted as a function of 2D wave vector in 7‐ML Cs thin films on CVS. Red dots and dashed lines indicate the experimental Fermi wave vectors and Fermi surfaces, respectively, for the outermost QWSs. d) ARPES intensity measured along the ΓM high‐symmetry line in 7‐ML Cs thin films on CVS. e) Top view of fcc K(111), Rb(111), and Cs(111) layers. Black dashed lines and colored solid lines compare the size of hexagonal lattice between AVS and alkali metals.

Here we discuss the mechanism of growth of single‐crystalline Cs films on CVS. A key aspect for understanding this mechanism lies in the presence of a hexagonal Cs layer embedded in a CVS crystal (see the top view of the crystal in Figure 1b). This Cs layer has the same lattice symmetry as that of the (111)‐oriented Cs crystal with the fcc or body‐centered cubic (bcc) structure (**Figure** **3**a), and is exposed on the cleaved surface. Therefore, a (111)‐oriented Cs film is expected to be grown when the lattice constant reasonably matches that of the Cs layer in CVS. To examine this possibility, we calculated the total energy for the formation of Cs(111) films with varying the in‐plane lattice constant *a* for the bcc and fcc structures (see Figure 3a for the definition of *a*). The slab thickness in the calculations was fixed at 7 MLs, identical to that of the thinnest film which we obtained. The calculated total energy plotted as a function of *a* (Figure 3b) indicates that the equilibrium lattice constant for the bcc phase is *a* = 8.60 Å (see gray dots in Figure 3b), which is significantly larger than and further incommensurate to that of the Cs layer in CVS (5.49 Å; red dashed line). By contrast, the equilibrium lattice constant for the fcc phase is *a* = 5.48 Å (blue dots), showing an almost perfect coincidence with that of the Cs layer in CVS. Thus, it is natural to conclude that fcc Cs(111) is energetically more stable than bcc Cs(111) on CVS. To verify this point experimentally, we estimated lattice constants from the ARPES results (note that other structural characterizations, such as x‐ray diffraction, were unfeasible due to the critical requirement of low temperatures to keep the single crystalline phase). Since the momentum location of the M point in the hexagonal Brillouin zone is given by | ***k***_M_| = 2π/√3*a* (where *a* is the in‐plane lattice constant), the *a* value can be deduced by determining | ***k***_M_| in the ARPES results. Using this method, which was successfully applied to various compounds,^[^ ^40^ ^]^ we determined | ***k***_M_| in 7‐ML Cs film as 0.666 ± 0.012 Å^−1^ from the symmetric band structure with respect to the M point of the outermost QWSs (Figure 3c,d). The resulting value of *a* = 5.45 ± 0.10 Å reasonably agrees with *a* = 5.48 Å calculated for fcc Cs, whereas it is substantially smaller than *a* = 8.60 Å for bcc Cs, confirming the formation of the fcc phase.

We also calculated the total energy for 7‐ML fcc Rb(111) and K(111) and obtained the equilibrium lattice constant of 5.04 Å (magenta dots) and 4.70 Å (green dots), respectively. Figure 3e displays a comparison of the lattice size between fcc alkali metals (colored solid line) and bulk AVS (black dashed line) for K (left), Rb (middle), and Cs (right). As recognized from Figure 3e, the equilibrium lattice constant of fcc alkali metals gradually increases with the size of atomic radius, whereas that in AVS remains almost constant within ≈0.02 Å. As a result, while the lattice mismatch is negligibly small (only 0.2%) for Cs, it becomes ≈8% for Rb and ≈14% for K. This alkali‐metal‐dependent lattice match/mismatch would account for the experimental results that Cs and Rb films are successfully grown while K films were difficult. Specifically, the tensile strain from AVS is too large to stabilize the fcc K structure on its surface.

The fabrication of fcc Cs and Rb films is intriguing for three reasons. First, it contrasts with the unstable nature of the fcc phase in their bulk crystals at ambient pressure. It is well known that the stable phase in bulk alkali metals takes the bcc structure, while the fcc phase is typically realized only under high pressure, for instance, exceeding 2.4 GPa for Cs.^[^ ^41^, ^42^ ^]^ Second, this metastable fcc phase appears to be stabilized by the application of an effectively large lattice strain in the “quasi‐homoepitaxial” growth process. Namely, the fabrication of Cs films on the Cs layer of CVS (or the Rb variant of these) is analogous to homoepitaxy. While homoepitaxy is often employed to produce high‐quality thin films, it generally prevents the introduction of lattice strain due to the essential similarity between the film and substrate materials. By contrast, in the present case, the lattice strain is inherently encoded in the Cs layer within CVS and transferred to the overlayer Cs film. Notably, the in‐plane lattice constant *a* of the hexagonal Cs layer in CVS (5.49 Å) is more than 30% smaller than that of bcc Cs at ambient pressure (8.60 Å). It is remarked that the strain as large as 30% is substantial even compared to cases of heteroepitaxial growth, in which applying strain is generally easier than homoepitaxy (see Table S1, Supporting Information). This suggests that the atomic layer structure embedded in the bulk crystal serves as a structural template to effectively introduce a large lattice strain through quasi‐homoepitaxial growth, since the atomic arrangement in bulk crystals is significantly influenced by neighboring atoms and deviates from that in elemental materials. Lastly, it is worth noting that superconductivity has been reported in pressurized bulk alkali metals.^[^ ^42^, ^43^, ^44^, ^45^ ^]^ Therefore, the fabricated Cs and Rb films provide a fertile ground to explore exotic properties including superconductivity, and also stimulate a comparative study on the origin of superconductivity in the fcc phase.

While we have discussed the formation of alkali‐metal films thus far, one might be interested in their influence on the band structure and physical properties of AVS. Since the band structure of AVS becomes inaccessible by ARPES after fabricating Cs overlayers, we carried out first‐principles calculations and found that the band structure of AVS may be insensitive to the formation of the epitaxial alkali‐metal overlayer, in contrast to the disordered case.^[^ ^24^ ^]^ Details are summarized in Supporting Information.

### 2.4 Evaluation of Many‐Body Interactions

**Figure 4:**
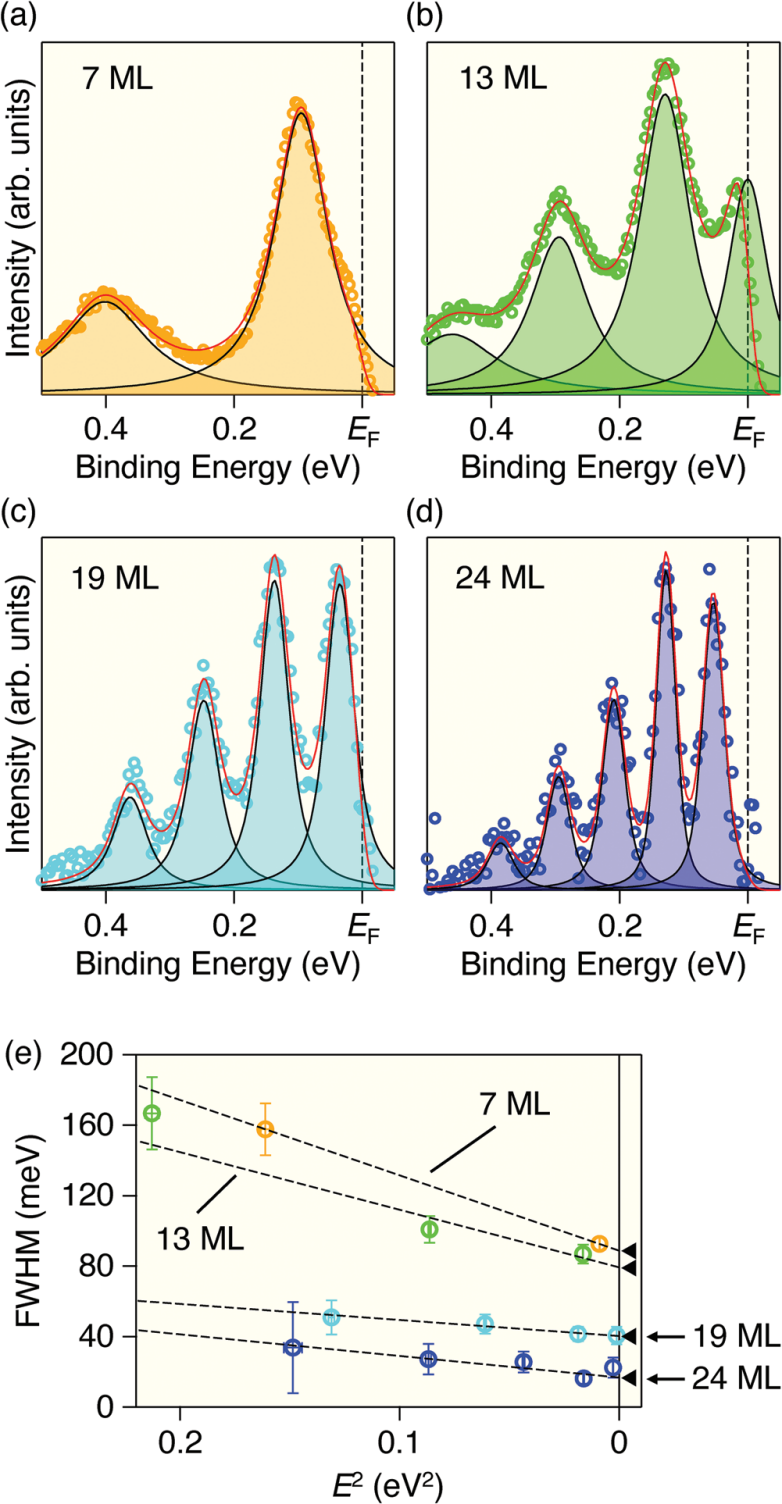
a–d) ARPES spectra at the Γ point for 7‐, 13‐, 19‐, and 24‐ML Cs films, respectively (circles; same as the data in Figure 2c but a linear background is subtracted), together with the result of multi‐peak fittings (red curve). Fitting result of each peak is indicated by black curve. e) The full width at half maximum (FWHM) of each Lorentzian in (a–d), plotted against *E* ^2^.

Another important characteristic of Cs thin films grown on CVS is the relatively wide tunability of film thickness. We note that a recent study reported that single‐crystalline fcc Cs thin films are stabilized on Cs‐intercalated bilayer graphene due to the adhesion energy between Cs and the bilayer graphene substrate.^[^ ^46^ ^]^ However, in this heterostructure, only 3 ML Cs film has been obtained experimentally so far.^[^ ^46^ ^]^ In contrast, we have successfully controlled the film thickness up to at least 30 MLs (Figure 3). This expanded range of accessible film thickness provides an opportunity to investigate the evolution of physical properties and quasiparticle dynamics as a function of film thickness. As a representative example, we conducted numerical analysis of the inverse lifetime of the quasiparticle, which is proportional to the full width at half maximum (FWHM) of quasiparticle peak. Specifically, we fitted the ARPES spectra at the Γ point for 7‐, 13‐, 19‐, and 24‐ML films with multiple Lorentzian peaks (**Figure** **4**a–d; identical to the spectra displayed in Figure 2c but after subtracting a linear background to make it easier to compare spectral line shapes) and plotted the extracted FWHM against the square of binding energy, *E* ^2^ (Figure 4e). As seen in Figure 4e, the FWHM monotonically decreases with increasing film thickness. Also, it decreases with approaching *E*_F_ at each film thickness, roughly following a linear relationship with *E* ^2^. The FWHM is approximately given by |2ImΣ(*E*)|, where ImΣ(*E*) is the imaginary part of the self‐energy. The *E* ^2^ behavior of the FWHM suggests that |2ImΣ(*E*)| basically follows a phenomenological function assuming the Fermi liquid ground states,^[^ ^25^ ^]^ as given by

(1) |2ImΣ(E)|=Γimp+2βE2

where the first term is the contribution from the impurity scattering and the second term is the contribution from the electron‐electron scattering. We fitted the FWHM with Equation (1) and obtained the parameters (Γ_imp_, 2β) as (0.088 ± 0.004, 0.42 ± 0.10), (0.080 ± 0.006, 0.328 ± 0.08), (0.040 ± 0.004, 0.10 ± 0.08), (0.016 ± 0.004, 0.12 ± 0.10) for 7‐, 13‐, 19‐, and 24‐ML films, respectively. As recognized from these values and the systematic variation of intercepts in the fitted lines in Figure 4e (indicated by triangles), the Γ_imp_ decreases with increasing the film thickness, possibly due to the increase in the quality of film. Also, the 2β, defined as the slope of the fitted line in Figure 4e, exhibits larger values in thinner films (7 and 13 MLs) than in thicker films (19 and 24 MLs). This suggests the enhanced electron‐electron correlation with approaching the 2D limit. While there are many ARPES studies on QWSs, the observation of a thickness‐dependent electron correlation effect has been limited to a few systems, such as Bi^[^ ^29^ ^]^ and SrVO_3_.^[^ ^30^ ^]^ Therefore, the present observation would accelerate a comparative investigation of QWSs in various systems with distinct properties, which is crucial to deepen the understanding of many‐body interactions in QWSs. Notably, Cs thin films are suitable for such comparison because there are key differences in the characteristics of QWSs, such as i) Bi exhibiting complex fermiology compared to a simpler band structure in Cs, ii) several experiments pointing out the topologically nontrivial character of Bi, in contrast to the trivial nature of Cs, iii) SrVO_3_ is a *d*‐orbital system, whereas Cs is an *s*‐orbital system, and iv) SrVO_3_ showing a thickness‐dependent metal‐insulator transition.

## 3 Conclusion

We have demonstrated that the low‐temperature deposition of Cs and Rb atoms on kagome superconductors produces their single‐crystalline thin films with a wide tunable thickness range. The present process to fabricate single‐crystalline films is regarded as quasi‐homoepitaxial growth on the Cs/Rb layer embedded in the bulk CVS/RVS crystal with substantial compressive strain inside the crystal. Fabricated single‐crystalline Cs and Rb films were found to take the fcc structure in contrast to the bulk counterpart which takes the bcc structure at ambient pressure and transforms into the fcc one under high pressure. The present findings provide a wide range of opportunities to investigate the thickness‐dependent evolution of QWS, quasiparticle lifetime, related physical properties in the fcc phase, and their interaction with novel properties of kagome lattice. It is particularly interesting to carry out in situ transport measurements in the future.

## 4 Experimental Section

### Sample Fabrication and ARPES Measurements

High‐quality bulk AVS (A = K, Rb, and Cs) single crystals were synthesized with the self‐flux method.^[^ ^18^ ^]^ AVS was cleaved at *T* = 10–20 K in ultrahigh vacuum of < 1×10^−10^ Torr and immediately carried out the alkali‐metal dosing by using alkali‐metal dispensers (SAES Getters) as schematically shown in Figure 1c. It was noted that the formation of any ordered Cs films was not observed when Cs was deposited at *T* = 300 K; it simply results in electron doping in CVS.^[^ ^24^ ^]^ ARPES measurements were performed using MBS‐A1 analyzer at BL5U in UVSOR and Scienta‐Omicron SES2002 spectrometer at Tohoku University. Linearly polarized photons of *hν* = 56 and 106 eV were used at UVSOR, and a He discharge lamp (He Iα; *hν* = 21.218 eV) at Tohoku University. The energy resolution was set to be 50 meV at UVSOR and 25 meV at Tohoku University.

### First‐Principles Calculations

First‐principles band calculations were carried out by using the Quantum Espresso code package with generalized gradient approximation.^[^ ^47^, ^48^ ^]^ Ultrasoft pseudopotential was used in the calculations. The plane‐wave cutoff energy and the *k*‐point mesh were set to be 30 Ry and 14 × 14 × 1, respectively. For the slab calculations, the thickness of inserted vacuum layer was set to be more than 30 Å to prevent interlayer interaction.

## Conflict of Interest

The authors declare no conflict of interest.

## Supporting information

Supporting Information

## Data Availability

The data that support the findings of this study are available from the corresponding author upon reasonable request.
