# Supplementary material for: Quasi‐Homoepitaxial Growth of Highly Strained Alkali‐Metal Ultrathin Films on Kagome Superconductors
Source: Adv Sci (Weinh). 2024 Jun 3;11(29):2309003. doi: 10.1002/advs.202309003 (PMC11304331; doi:10.1002/advs.202309003)
Supplement: Supplementary file 1 — Supporting Information [file ADVS-11-2309003-s001.pdf]

ADVANCED SCIENCE
Open Access

Supporting Information

for *Adv. Sci.*, DOI 10.1002/advs.202309003

Quasi-Homoepitaxial Growth of Highly Strained Alkali-Metal Ultrathin Films on Kagome Superconductors

Takemi Kato, Kosuke Nakayama, Yongkai Li, Zhiwei Wang, Katsuaki Sugawara, Kiyohisa Tanaka, Takashi Takahashi, Yugui Yao and Takafumi Sato**

Supporting Information

Quasi-Homoepitaxial Growth of Highly Strained Alkali-Metal Ultrathin Films on Kagome Superconductors

Takemi Kato, Kosuke Nakayama, Yongkai Li, Zhiwei Wang, Katsuaki Sugawara, Kiyohisa Tanaka, Takashi Takahashi, Yugui Yao, and Takafumi Sato**

S1. Estimation of film thickness

The thickness of Cs thin films was estimated by comparing the energy separation of quantum well states (QWSs) in the angle-resolved photoemission spectroscopy (ARPES) results with calculations with varying thicknesses, following a common practice in ARPES studies. For example, when the second-derivative ARPES intensity of Figure 2a1 in the main text is compared with band structure calculations with 6–8 monolayers (MLs) (**Figure S1a–c**, respectively), the energy difference between the topmost and next QWSs best agrees with the calculation for 7 ML (Figure S1b), whereas there is a clear discrepancy for the calculations of 6 and 8 MLs (indicated by white arrows in Figure S1a,c) (note that the calculated bands in these figures were shifted in energy so that the energy position of the topmost QWS coincides with that of the ARPES data). Based on this comparison, the thickness was estimated to be 7 ML.

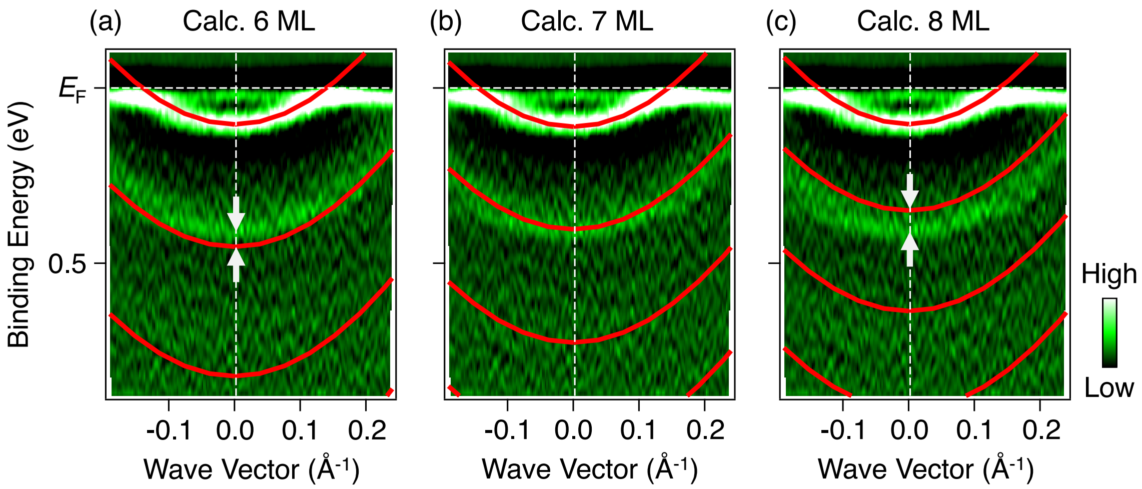

Figure S1. (a–c) Second-derivative ARPES intensity for Cs-deposited CsV_3Sb_5, reproduced from Figure 2b1 in the main text, together with calculated band structures for 6-ML, 7-ML, and 8-ML fcc Cs, respectively (red curves). White arrows highlight an energy difference between the experimental and calculated QWSs.

S2. Comparison of the size of epitaxial strain

In **Table S1**, we summarize typical examples of epitaxial thin films in which lattice strain has been successfully introduced.[1–22] In this table, the size of strain was calculated by $1-a_{Film}/a_{Bulk}$, where a_{Film} and a_{Bulk} are the in-plane lattice constants of epitaxial film and its bulk counterpart. One can see that the size of strain is usually less than 3%. The largest strain is limited to ~5% even with the heteroepitaxial growth technique. Compared with these values, the magnitude of strain, as large as 30%, in Cs thin films on CsV_3Sb_5 is substantially large.

Table S1: Size of strain applied to epitaxial thin films.

Film	Substrate	a_{Film} (Å)	a_{Bulk} (Å)	Strain (%)	Ref.
Cs	Cs (CsV_3Sb_5)	5.48	8.60	36.3	This work
$La_{2-x}Sr_xCuO_4$	$LaSrAlO_4$	3.76	3.78	0.6	[1]
$La_{2-x}Sr_xCuO_4$	$SrTiO_3$	3.80	3.78	-0.5	[1]
$Nd_{1.83}Ce_{0.17}CuO_x$	$YBa_2Cu_3O_{7-\delta}$	3.88	3.94	1.5	[2]
$SrRuO_3$	$SrTiO_3$	3.90	3.93	0.7	[3]
$SrRuO_3$	$DyScO_3$	3.94	3.93	-0.3	[3]
$SrRuO_3$	$TbScO_3$	3.96	3.93	-0.8	[3]
$SrRuO_3$	$GdScO_3$	3.95	3.93	-0.6	[3]
$SrRuO_3$	$SmScO_3$	3.99	3.93	-1.5	[3]
$La_{0.67}Sr_{0.33}MnO_3$	$LaAlO_3$	3.82	3.87	1.4	[4]
$La_{0.67}Sr_{0.33}MnO_3$	NdGaO3	3.86	3.87	0.3	[4]
$La_{0.67}Sr_{0.33}MnO_3$	$La_{0.3}Sr_{0.7}Al_{0.65}Ta_{0.35}O_3$	3.87	3.87	0.1	[4]
$La_{0.67}Sr_{0.33}MnO_3$	$SrTiO_3$	3.90	3.87	-0.8	[4]
$La_{0.7}Ca_{0.15}Sr_{0.15}MnO_3$	$0.67Pb(Mg_{1/3}Nb_{2/3})O_3$-$0.33PbTiO_3$	3.90	3.89	-0.4	[5]
$PbZr_{0.2}Ti_{0.8}O_3$	$SrRuO_3/SrTiO_3$	3.91	3.95	1.1	[6]
$SrTiO_3$	$(LaAlO_3)_{0.3}(Sr_2AlTaO_6)_{0.7}$	3.87	3.91	1.0	[7]
$LaCoO_3$	$SrLaAlO_4$	3.82	3.80	-0.5	[8]
$LaCoO_3$	$LaAlO_3$	3.81	3.80	-0.1	[8]
$LaCoO_3$	$SrLaGaO_4$	3.86	3.80	-1.5	[8]
$LaCoO_3$	$(LaAlO_3)_{0.3}(Sr_2AlTaO_6)_{0.7}$	3.87	3.80	-1.8	[8]
$LaCoO_3$	$SrTiO_3$	3.89	3.80	-2.4	[8]
V_2O_3	α-Al_2O_3	4.99	4.95	-0.7	[9]
RuO_2	TiO_2	2.96	3.11	4.8	[10]
VO_2	TiO_2	4.69	4.56	-3.0	[11]

LaO	$YAlO_3$	5.20	5.25	0.9	[12]
LaO	$LaSrAlO_3$	5.30	5.25	-1.0	[12]
TiO	α-Al_2O_3	4.16	4.18	0.3	[13]
MgO	Ag	4.07	4.20	3.1	[14]
$BaFe_2As_2$	$SrTiO_3$	4.04	3.96	-2.0	[15]
Fe_2VSi	MgO	5.69	5.67	-0.3	[16]
Fe_2VSi	$MgAl_2O_4$	5.71	5.67	-0.7	[16]
FeSe	CaF_2	3.73	3.78	1.2	[17]
FeSe	$SrTiO_3$	3.90	3.76	-3.7	[18]
VSe_2	α-Al_2O_3	3.49	3.35	-4.2	[19]
$PdTe_2$	$SrTiO_3$	4.00	4.04	1.0	[20]
$MnTe_2$	Si	4.10	4.16	1.4	[21]
Bi	Bi_2Te_3	4.39	4.54	3.3	[22]

S3. Band structure calculations in the presence of epitaxial Cs overlayer

To discuss how epitaxial Cs films influence the electronic band structure and physical property of CVS, we conducted first-principles calculations of the band structure before and after the formation of epitaxial Cs film (the corresponding slabs are depicted in **Figure S2a and S2b**, respectively). A comparison of the calculated results in Figure S2c reveals that the CVS-derived V 3*d* bands dominating the band structure around the K and M points are almost unchanged. In addition, while the CVS-derived Sb 5*p* bands near the Γ point show a finite energy shift due to the hybridization with the QWSs of the Cs film, their Fermi wave vectors are almost unchanged. These findings suggest very little charge transfer between the epitaxial Cs film and CVS, in sharp contrast to the observation of electron doping from a disordered Cs overlayer to CVS. Since the energy position of the saddle point and the Sb 5*p* band filling are the key band parameters linked to the CDW and superconductivity in CVS, the formation of the epitaxial Cs overlayer may not alter these properties.

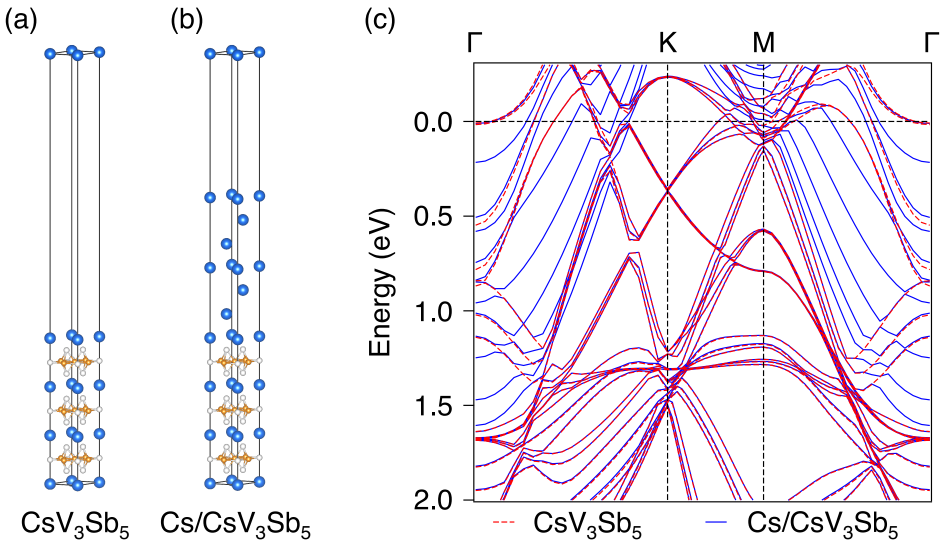

Figure S2. (a,b) Slabs of CsV_3Sb_5 without and with epitaxial Cs overlayer, respectively. (c) Calculated band structures using the slabs in (a,b).

References

[1] J.-P. Locquet, J. Perret, J. Fompeyrine, E. Mächler, J. W. Seo, G. Van Tendeloo, *Nature* **1998**, *394*, 453–456.

[2] A. Gupta, R. Gross, E. Olsson, A. Segmüller, G. Koren, C. C. Tsuei, *Phys. Rev. Lett.* **1990**, *64*, 3191.

[3] Y. K. Wakabayashi, S. Kaneta-Takada, Y. Krockenberger, Y. Taniyasu, H. Yamamoto, *ACS Appl. Electron. Mater.* **2021**, *3*, 2712–2719.

[4] S. K. Chaluvadi, F. Ajejas, P. Orgiani, S. Lebargy, A. Minj, S. Flament, J. Camarero, P. Perna, L. Méchin, *J. Phys. D: Appl. Phys.* **2020**, *53*, 375005.

[5] R. K. Zheng, H.-U. Habermeier, H. L. W. Chan, C. L. Choy, H. S. Luo, *Phys. Rev. B* **2009**, *80*, 104433.

[6] H. N. Lee, S. M. Nakhmanson, M. F. Chisholm, H. M. Christen, K. M. Rabe, D. Vanderbilt, *Phys. Rev. Lett.* **2007**, *98*, 217602.

[7] K. Ahadi, L. Galletti, Y. Li, S. Salmani-Rezaie, W. Wu, S. Stemmer, *Sci. Adv.* **2019**, *5*, eaaw0120.

[8] D. Fuchs, E. Arac, C. Pinta, S. Schuppler, R. Schneider, H. v. Löhneysen, *Phys. Rev. B* **2008**, *77*, 014434.

[9] H. Schuler, S. Klimm, G. Weissmann, C. Renner, S. Horn, *Thin Solid Films* **1997**, *299*, 119–124.

[10] J. P. Ruf, H. Paik, N. J. Schreiber, H. P. Nair, L. Miao, J. K. Kawasaki, J. N. Nelson, B. D. Faeth, Y. Lee, B. H. Goodge, B. Pamuk, C. J. Fennie, L. F. Kourkoutis, D. G. Schlom, K. M. Shen, *Nat. Commun.* **2021**, *12*, 59.

[11] B. Chen, G. Kim, H. J. Cho, H. Ohta, *Adv. Electron. Mater.* **2022**, *8*, 2100687.

[12] K. Kaminaga, D. Oka, T. Hasegawa, T. Fukumura, *J. Am. Chem. Soc.* **2018**, *140*, 6754–6757.

[13] C. Zhang, F. Hao, G. Gao, X. Liu, C. Ma, Y. Lin, Y. Yin, X. Li, *npj Quant. Mater.* **2017**, *2*, 2.

[14] S. Valeri, S. Altieri, A. di Bona, P. Luches, C. Giovanardi, T.S. Moia, *Surf. Sci.* **2002**, *507–510*, 311–317.

[15] J. Engelmann, V. Grinenko, P. Chekhonin, W. Skrotzki, D. V. Efremov, S. Oswald, K. Iida, R. Hühne, J. Hänisch, M. Hoffmann, F. Kurth, L. Schultz, B. Holzapfel, *Nat. Commun.* **2013**, *4*, 2877.

[16] N. Fukatani, K. Ueda, H. Asano, *J. Appl. Phys.* **2011**, *109*, 073911.

[17] F. Nabeshima, Y. Imai, M. Hanawa, I. Tsukada, A. Maeda, *Appl. Phys. Lett.* **2013**, *103*, 172602.

[18] S. Tan, Y. Zhang, M. Xia, Z. Ye, F. Chen, X. Xie, R. Peng, D. Xu, Q. Fan, H. Xu, J. Jiang, T. Zhang, X. Lai, T. Xiang, J. Hu, B. Xie, D. Feng, *Nat. Mater.* **2013**, *12*, 634–640.

[19] D. Zhang, J. Ha, H. Baek, Y.-H. Chan, F. D. Natterer, A. F. Myers, J. D. Schumacher, W. G. Cullen, A. V. Davydov, Y. Kuk, M. Y. Chou, N. B. Zhitenev, J. A. Stroscio, *Phys. Rev. Mater.* **2017**, *1*, 024005.

[20] C. Liu, C.-S. Lian, M.-H. Liao, Y. Wang, Y. Zhong, C. Ding, W. Li, C.-L. Song, K. He, X.-C. Ma, W. Duan, D. Zhang, Y. Xu, L. Wang, Q.-K. Xue, *Phys. Rev. Mater.* **2018**, *2*, 094001.

[21] S. Lu, K. Peng, P. D. Wang, A. X. Chen, W. Ren, X. W. Fang, Y. Wu, Z. Y. Li, H. F. Li, F. Y. Cheng, K. L. Xiong, J. Y. Yang, J. Z. Wang, S. A. Ding, Y. P. Jiang, L. Wang, Q. Li, F. S. Li, L. F. Chi, *Chin. Phys. B* **2021**, *30*, 126804.

[22] T. Hirahara, N. Fukui, T. Shirasawa, M. Yamada, M. Aitani, H. Miyazaki, M. Matsunami, S. Kimura, T. Takahashi, S. Hasegawa, K. Kobayashi, *Phys. Rev. Lett.* **2013**, *109*, 227401.
